# Cytotoxicity of guanine-based degradation products contributes to the antiproliferative activity of guanine-rich oligonucleotides†
†Electronic supplementary information (ESI) available: Experimental details and supplementary figures. See DOI: 10.1039/c4sc03949a


**DOI:** 10.1039/c4sc03949a

**Published:** 2015-04-07

**Authors:** Nan Zhang, Tao Bing, Xiangjun Liu, Cui Qi, Luyao Shen, Linlin Wang, Dihua Shangguan

**Affiliations:** a Beijing National Laboratory for Molecular Sciences , Key Laboratory of Analytical Chemistry for Living Biosystems , Institute of Chemistry , Chinese Academy of Sciences , Beijing , 100190 , China . Email: sgdh@iccas.ac.cn ; Fax: +86-10-62528509 ; Tel: +86-10-62528509; b University of the Chinese Academy of Sciences , Beijing 100049 , China

## Abstract

Guanine-rich oligonucleotides (GROs) have attracted considerable attention as anticancer agents, because they exhibit cancer-selective antiproliferative activity and can form G-quadruplex structures with higher nuclease resistance and cellular uptake. Recently, a GRO, AS1411 has reached phase II clinical trials for acute myeloid leukemia and renal cell carcinoma. The antiproliferative activity of GROs has been associated with various protein targets; however the real mechanisms of action remain unclear. In this study, we showed evidence that antiproliferative activity of GROs (including AS1411) is mainly contributed by the cytotoxicity of their guanine-based degradation products, such as monophosphate deoxyguanosine (dGMP), deoxyguanosine (dG) and guanine. The GROs with lower nuclease resistance exhibited higher antiproliferative activity. Among nucleotides, nucleosides and nucleobases, only guanine-based compounds showed highly concentration-dependent cytotoxicity. Our results suggest that it is necessary to reconsider the cancer-selective antiproliferative activity of GROs. Since guanine-based compounds are endogenous substances in living organisms, systematic studies of the cytotoxicity of these compounds will provide new information for the understanding of certain diseases and offer useful information for drug design.

## Abstract 2

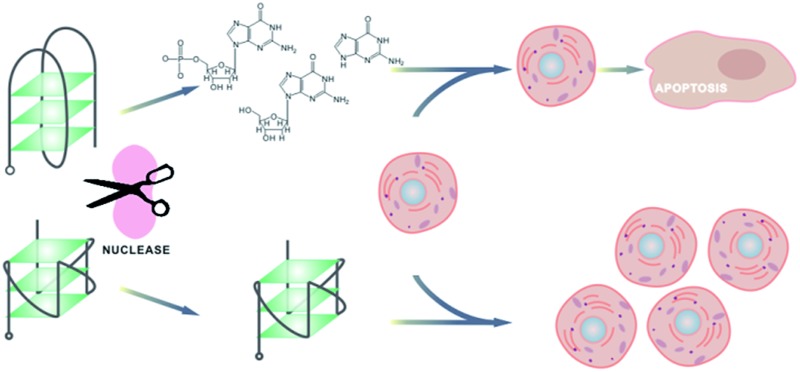
Guanine-rich oligonucleotides with lower nuclease resistance exhibited higher antiproliferative activity; guanine-based compounds showed highly concentration-dependent cytotoxicity.

## Introduction

Exploration of oligonucleotides as therapeutic agents has attracted extensive efforts over the last two decades. Although many strategies, such as antisense oligonucleotides, small interfering RNA,^1^ aptamers,^2^–^6^ immunostimulatory CpG^7^ and molecular decoys,^8^ have exhibited considerable therapeutic promise, the *in vivo* usefulness of oligonucleotide-based medicines is limited by their poor cellular internalization/trafficking^9^,^10^ and their susceptibilities to degradation by various nucleases present in almost every biological fluid.^1^ Recently, guanine-rich oligonucleotides (GROs) have attracted considerable interest because they can form G-quadruplex structures, a characteristic secondary structure that is composed of planar arrangements of four G-bases stabilized by eight Hoogsteen hydrogen bonds (known as a G-quartet).^11^ Compared to other native oligonucleotides, G-quadruplexes are found to have increased nuclease resistance and enhanced cellular uptake.^12^–^15^ Many GROs have been reported to have rather distinct biological activities, such as anticoagulant,^16^ antiviral^17^–^19^ and cancer-selective antiproliferative activity.^20^–^25^ Recently, GRO libraries (random sequences) were also reported to have strong antiproliferative activity, which suggests that the antiproliferative activity may be a general feature of certain GROs.^26^

Different from the antisense oligonucleotides that hybridize to target nucleic acids, the activities of GROs are considered to arise from binding to protein targets,^12^,^27^,^28^ thus, many mechanisms of antiproliferative activity of GROs have been proposed.^29^–^31^ However, the real molecular basis of the antiproliferative activity of GROs remains unclear.

An important achievement of the therapeutic oligonucleotides is AS1411, a GRO that has reached phase II clinical trials for acute myeloid leukemia and renal cell carcinoma.^32^ AS1411 is a G-quadruplex-forming oligodeoxynucleotide, which has been found to exhibit antiproliferative activity in various cancer cell types and exhibit antitumor activity in several animal xenograft models without toxic effect.^12^,^33^,^34^ The molecular target of AS1411 is considered to be nucleolin, a multifunctional protein overexpressed in cytoplasm and on the cell surface of many tumor types, thus it has been widely used as a nucleolin-binding aptamer in cancer-cell-specific drug delivery and cancer cell imaging.^35^–^38^ The cellular uptake of AS1411 was previously considered to be mediated by surface nucleolin (as receptor), and then was also found to be mediated through micropinocytosis in some cell types. In the micropinocytosis pathway, nucleolin was not required for initial AS1411 uptake but was necessary for induced micropinocytosis.^39^ Although some mechanisms of action of AS1411 have been proposed, such as inhibition of NF-κB activation, S-phase cell cycle arrest, derepression of some PRMT5 target genes, and reduction of bcl-2 expression; and nucleolin has also been found to be involved in these mechanisms,^30^,^34^,^40^,^41^ the exact role of nucleolin and the real mechanism of AS1411 are not completely understood.

In a previous study, we found that intramolecular G-quadruplex oligonucleotides with parallel structures have a general binding activity to many cell lines. Some of these G-quadruplexes exhibited antiproliferative activity independent of their cellular binding.^42^ In our further study on the relationship of antiproliferative activity and G-quadruplex structures, we found that the antiproliferative activity of GROs might be contributed by the cytotoxicity of their guanine-based degradation products. In this paper, we show the evidence to support this presumption.

## Results

### Antiproliferative activity of GROs

Our original experimental design was to investigate the relationship between antiproliferative activity and G-quadruplex structures, thus we designed a group of GROs as CTG_3_H_*x*_G_3_H_*x*_G_3_H_*x*_G_3_A (Table 1), where H_*x*_ are loops of different length, H represents base A, C or T, and *x* represents the number of bases within the limit of 1–3. This kind of oligonucleotide is considered to form G-quadruplexes with different loops. G-quadruplexes with single-base loops usually adopt a parallel structure and have a high thermostability; as the loop length increases, G-quadruplexes prefer to adopt an antiparallel structure, hybrid or mixed parallel/antiparallel structure with less thermostability.^43^–^45^ We also synthesized two GROs with non-nucleotide loops: propyl loops (C3-loop) and triethylene glycol loops (S9-loop). AS1411 was also synthesized as the positive control. The Circular Dichroism spectra experiment confirmed that these oligonucleotides could fold into G-quadruplexes in phosphate buffered saline (PBS) (Fig. S1†). Among them, the oligonucleotides with single-base loops and non-nucleotide loops (T-loop, C-loop, A-loop, H-loop, C3-loop and S9-loop) exhibited strong characteristic signals of parallel G-quadruplexes, suggesting that the formed G-quadruplexes were highly stable.^43^–^45^

**Table 1:** The sequences of oligonucleotides used in this work

	Oligo	Sequence (from 5′ to 3′)
G-quadruplex	T-loop	CTGGGTGGGTGGGTGGGA
	C-loop	CTGGGCGGGCGGGCGGGA
	A-loop	CTGGGAGGGAGGGAGGGA
	H-loop	CTGGGHGGGHGGGHGGGA
	TT-loop	CTGGGTTGGGTTGGGTTGGGA
	CC-loop	CTGGGCCGGGCCGGGCCGGGA
	AA-loop	CTGGGAAGGGAAGGGAAGGGA
	HH-loop	CTGGGHHGGGHHGGGHHGGGA
	TTT-loop	CTGGGTTTGGGTTTGGGTTTGGGA
	HHH-loop	CTGGGHHHGGGHHHGGGHHHGGGA
	AS1411	GGTGGTGGTGGTTGTGGTGGTGGTGG
	C3-loop	CTGGGXGGGXGGGXGGGA, X = propyl
	S9-loop	CTGGGYGGGYGGGYGGGA, Y= triethylene glycol
Non-G-quadruplex	H-G4	CTGGGTTGGG
	C-control	CTCCCTTCCCTTCCCTTCCCA
	G-control	CTTTTGGTTTGGTTTGGTTTA

**Fig. 1:**
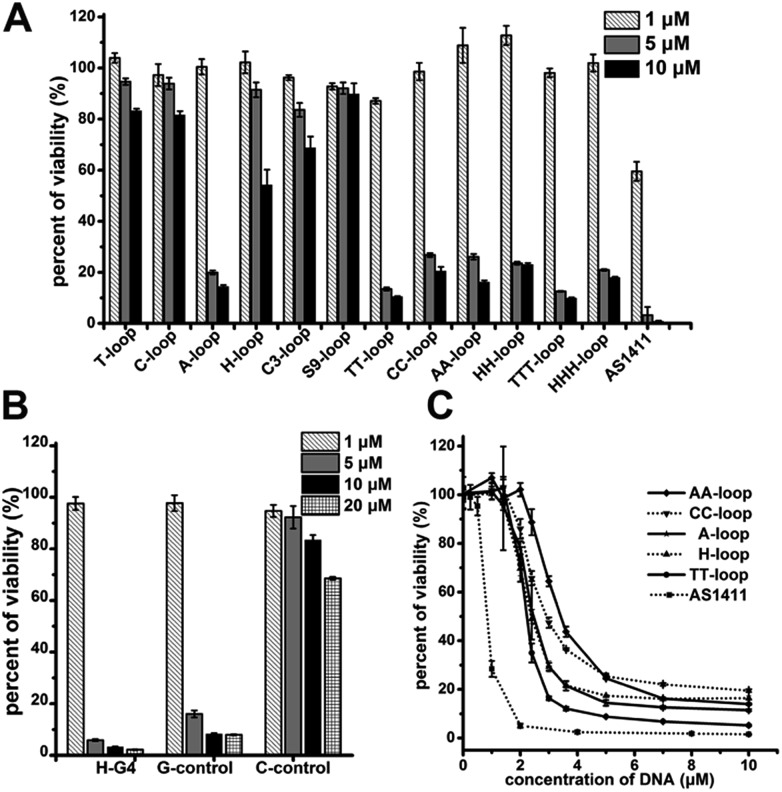
Antiproliferative activities of oligonucleotides on Jurkat E6-1 cells. (A) Antiproliferative activities of G-quadruplex oligonucleotides. (B) Antiproliferative activities of non-G-quadruplex oligonucleotides. (C) Dose–response antiproliferative effect of GROs. Cell viability was measured by CCK-8 assay after treating cells for 96 h. Bars represent mean ± SEM, *n* = 3.

The cell proliferation inhibition by these sequences was measured using Jurkat E6-1 cell line (human acute T cell leukemia) because G-quadruplexes do not bind this cell line,^42^ which may eliminate the influence of different binding affinity to cells of different sequences. All the G-quadruplexes with two- or three-base loops showed a strong antiproliferative effect on Jurkat E6-1 cells (>70% growth inhibition) at concentrations of 5 μM and 10 μM (Fig. 1A). However, among the G-quadruplexes with single-base loops or non-nucleotide loops, only the A-loop showed a strong antiproliferative effect on Jurkat E6-1 at the same concentration; and the H-loop showed a weak antiproliferative effect, *i.e.* ∼40% growth inhibition at 10 μM. The positive control, AS1411 showed a very strong antiproliferative effect at concentrations of 5 μM and even ∼40% growth inhibition at 1 μM. These results imply that a stable G-quadruplex structure might not be essential for the antiproliferative activity of GROs. Therefore we further tested the antiproliferative activity of three control oligonucleotides that cannot form intramolecular G-quadruplexes: H-G4 (3′ half of TT-loop), C-control (without G bases), and G-control (only three G2 tracts) (Table 1). C-control did not show a strong antiproliferative effect even at concentrations of 20 μM; H-G4 and G-control exhibited a strong antiproliferative effect at concentrations of 5 μM (Fig. 1B), suggesting that G-base is necessary for the antiproliferative activity, but the G-quadruplex structure is not necessary. The dose dependent effect of the oligonucleotides that showed an antiproliferative effect was further measured (Fig. 1C). The IC_50_ values (the concentration that causes 50% growth inhibition) of these G-quadruplex oligonucleotides were estimated in the range of 2.1–3.2 μM, and the IC_50_ value of AS1411 was 0.8 μM, which is consistent with that previously reported.^12^

### Nuclease resistance of GROs

**Fig. 2:**
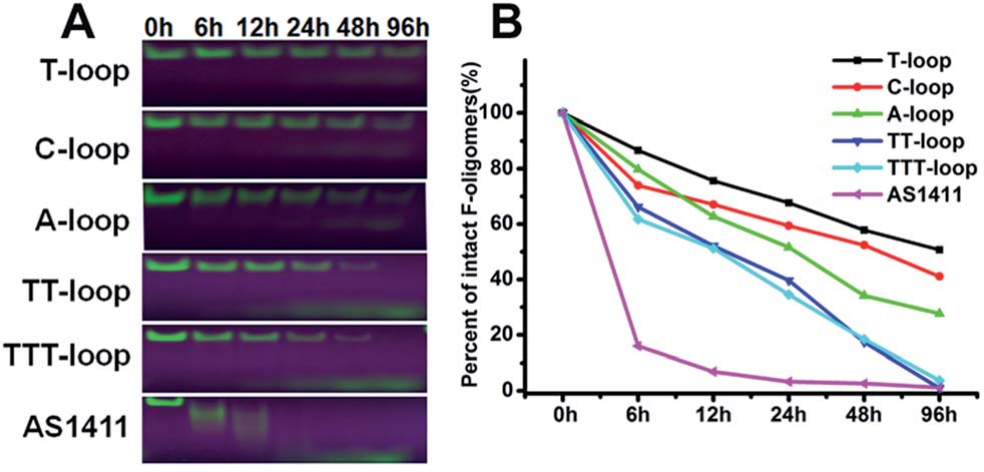
Nuclease resistance of GROs. (A) Denaturing-polyacrylamide gel (20%) electrophoresis assay of 5′-fluorescein-labeled GROs (10 μM) after incubated in RPMI 1640 medium with 10% FBS at 37 °C for different time, gels were exposed under UV light and photographed. (B) Degradation curves of GROs, data were extracted from A.

It has been reported that compact intramolecular G-quadruplexes have high nuclease resistance.^13^ Our previous studies have shown that a very close analog of the T-loop has a much stronger nuclease resistance than AS1411;^42^ T-loop and C-loop have higher thermostability (melting temperature (*T*_m_) > 80 °C) than A-loop and TT-loop (*T*_m_: 65 and 61 °C).^45^ These results together with the above results imply that the degradation of GROs by nuclease may play an important role in their antiproliferative activity. Therefore we compared the nuclease resistance of these sequences in cell culture media (including 10% fetal bovine serum (FBS)) by gel electrophoresis (Fig. 2). T-loop, C-loop exhibited strong nuclease resistance, the fluorescence of intact oligonucleotides was still visible after 96 h, the half-life (*T*_1/2_) was estimated to be 96 and 52 h based on the decrease of fluorescence intensity of intact GROs. A-loop exhibited a medium level of nuclease resistance (*T*_1/2_, ∼25 h); TT-loop and TTT-loop showed weaker nuclease resistance (*T*_1/2_, ∼12–15 h), almost no intact oligonucleotides were observed after 96 h. Among these tested GROs, AS1411 showed the weakest nuclease resistance (*T*_1/2_, ∼2 h), most of them were digested in 6 h. The smear bands of AS1411 at 6 and 12 h suggest the progressive degradation of AS1411 from 3′-end (5′-Fluorescein-label). Other GROs did not show smear bands and only showed a low band at the longer time points, which may be due to the higher stability of these GROs that resulted in a very small amount of progressively degraded GROs or the degradation occurring at the FAM label.^46^ Comparing the antiproliferative activity and nuclease resistance of these GROs, a negative correlation was found (Fig. S2†), suggesting that the antiproliferative effects of GROs on Jurkat E6-1 cells may relate to their degradation products.

### Antiproliferative activity of guanine-based compounds

**Fig. 3:**
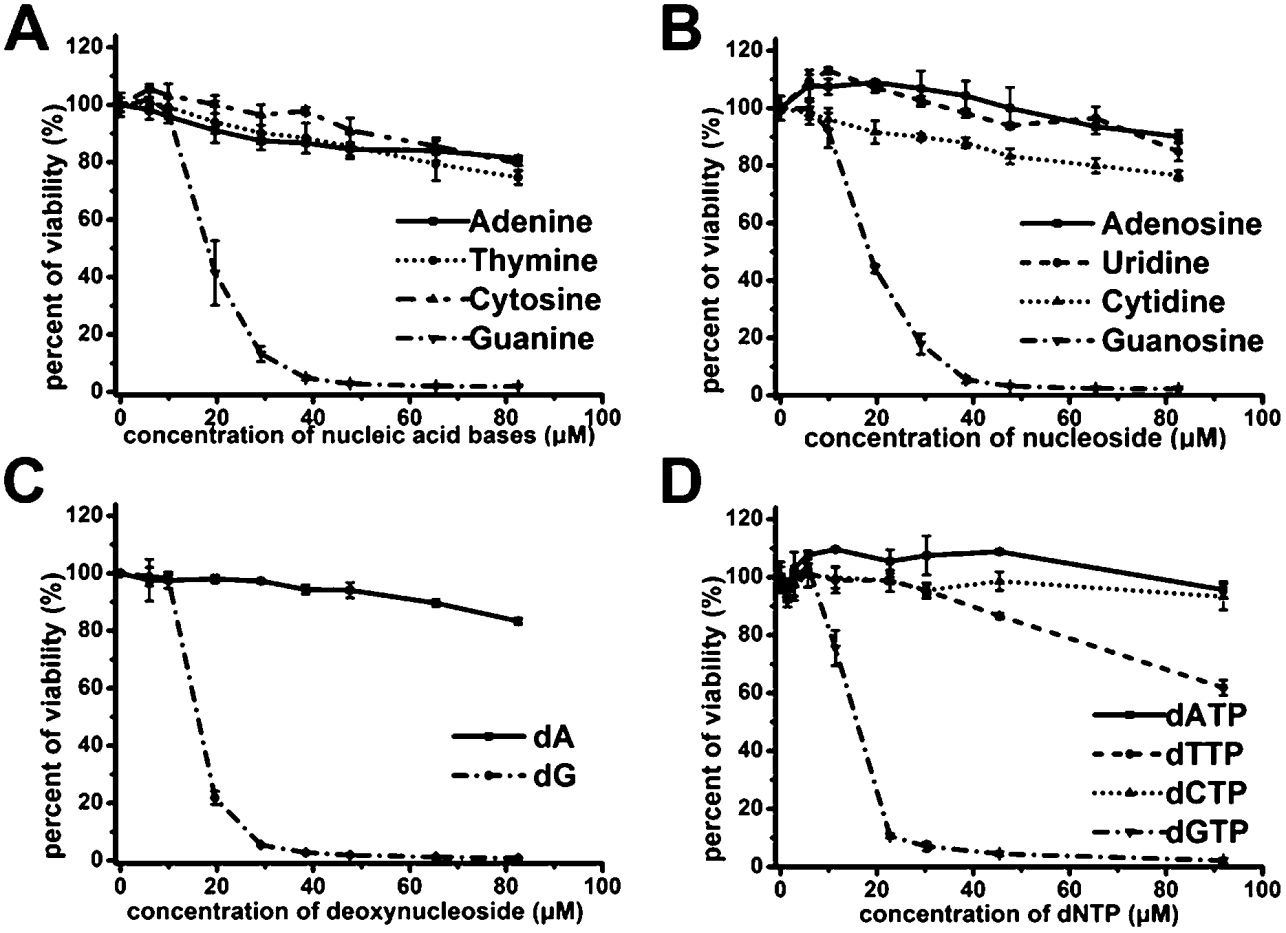
Antiproliferative activities of nucleobases, nucleosides and deoxy-ribonucleosides on Jurkat E6-1 cells. (A) nucleobases. (B) nucleosides. (C) deoxynucleosides. (D) dNTP. Cell viability was measured by CCK-8 assay after treating cells for 96 h. Bars represent mean ± SEM, *n* = 3.

In order to demonstrate above hypothesis, we tested the antiproliferative effect of nucleobases, nucleosides, deoxynucleosides, and deoxynucleoside triphosphate (dNTP) (Fig. 3A–D). Among these compounds, only guanine-based compounds (guanine, guanosine, deoxyguanosine (dG) and dGTP) showed a strong antiproliferative effect on Jurkat E6-1 cells, the IC_50_ were estimated to be in the range of 14–18 μM, and other nucleobase-related compounds did not exhibit significant antiproliferative effect, which further suggest that the guanine-based degradation products may contribute to the antiproliferative effects of GROs.

### Effects of GROs, dA and dG on different cell lines

**Fig. 4:**
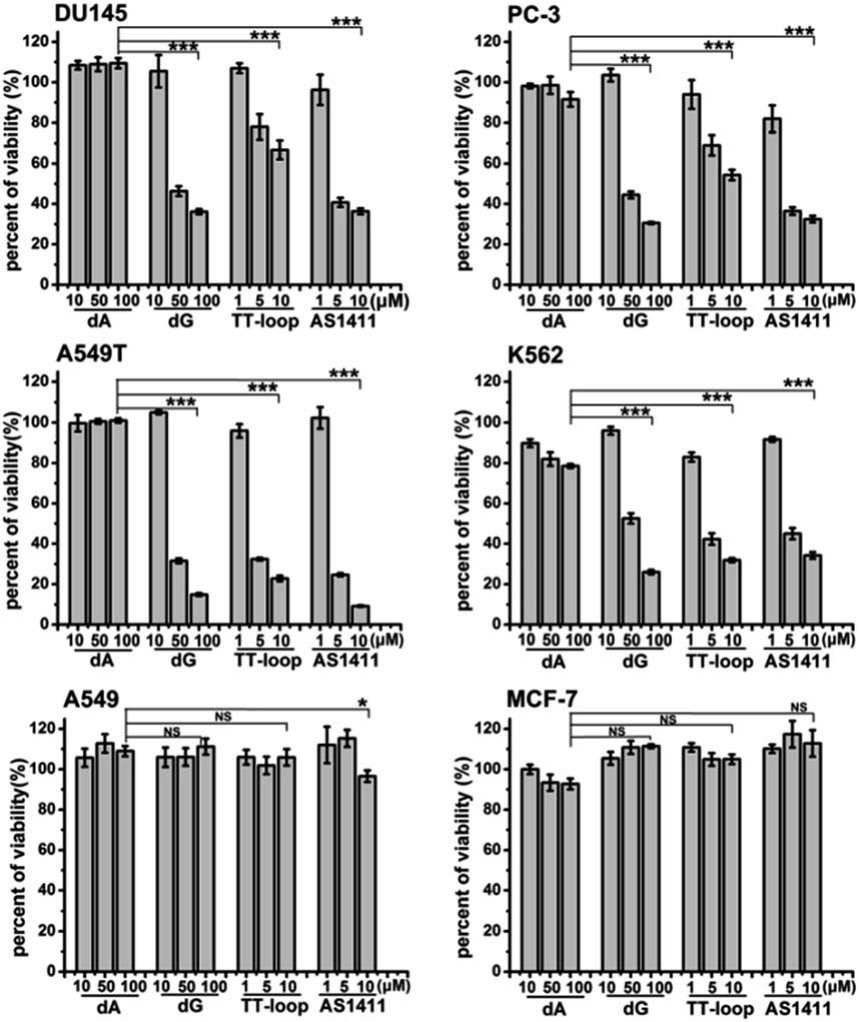
Antiproliferative activities of dA, dG, TT-loop and AS1411 on different cancer cell lines. Cell viability was measured by CCK-8 assay after treating cells for 96 h. Bars represent mean ± SEM, *n* = 3. The statistical significant differences between dA (100 μM) and dG (100 μM), TT-loop (10 μM) or AS1411 (10 μM) were calculated by IBM SPSS Statistic 20; *P < 0.05, ***P < 0.001 (*t*-test), NS, not significant.

The above antiproliferative effects were measured with Jurkat E6-1 cells. In order to demonstrate whether GROs and guanine-base compounds have a similar effect to other cell lines, we tested the proliferative inhibition effect of TT-loop, AS1411, deoxyadenosine (dA) and dG on six different cancer cell lines (A549, A549T, MCF-7, DU145, PC-3 and K562, see ESI† about cell lines) (Fig. 4). dA did not show any significant effect on all the tested cell lines. TT-loop, AS1411 and dG showed parallel effects on all the tested cell lines, *i.e.* they did not show significant antiproliferative effect on A549 and MCF-7 cell lines and showed significant antiproliferative effect on other cell lines, which implies that the GROs and dG may have the same mechanism of action; in other words, the cell growth inhibition by GROs may not be due to the whole oligonucleotide or G-quadruplex structure, and may be due to the action of their degradation products, such as dGMP, dG and guanine. These results may also explain our previous findings that the growth inhibition effect of G-quadruplexes was independent of their cellular binding.^42^

### Detection of guanine-based degradation products of GROs in serum

**Fig. 5:**
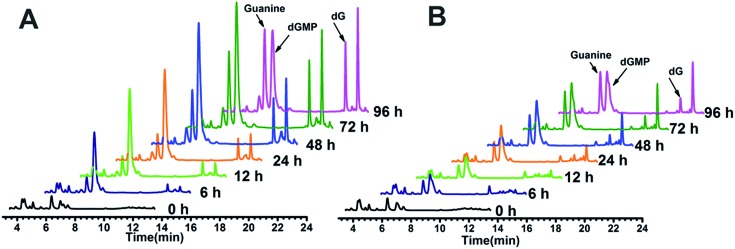
HPLC analysis of guanine-based degradation products of AS1411 (A) and TT-loop (B) in 10% serum. The peaks of dGMP, dG and guanine were confirmed by comparison with the standard compounds (Fig. S3†); the peak after dG corresponding to a thymine-based degradation product (Fig. S4†).

The above hypothesis was based on the observation that guanine-based compounds and GROs have parallel antiproliferative activity. In order to confirm this hypothesis we further detected the guanine-based degradation products of AS1411 and TT-loop during the degradation process. AS1411 (10 μM) and TT-loop (10 μM) were incubated in PBS containing 10% FBS at 37 °C for different times, then the guanine-based degradation products were analyzed by HPLC. Three guanine-based compounds, dGMP, dG and guanine were detected in AS1411 reaction solution (Fig. 5A). A high concentration of dGMP was observed at 6 h (44 μM), which gradually increased until 72 h (88 μM) and then declined at 96 h (63 μM). dG was observed to continuously increase from 6 h (0.3 μM) to 72 h (17 μM), and was then maintained at this level until 96 h (16 μM). Guanine was observed to continuously increase from 24 h (9 μM) to 96 h (46 μM). These changes of guanine-based compounds agreed with the degradation process of GROs, *i.e.* from deoxyoligonucleotide to dGMP to dG to guanine. Approximately 27–74% of AS1411 (containing a total of 170 μM guanine) were converted to guanine-based compounds from 6 to 48 h. These three compounds were also detected in TT-loop reaction solution from 6 to 96 h, but their concentrations were lower than that in AS1411 solution (Fig. 5B), and approximately 12–40% of the TT-loop (totally containing 120 μM guanine) were converted to guanine-based compounds from 6 to 96 h, which may be mainly due to its higher nuclease resistance than AS1411. This set of results confirms that guanine-based compounds were indeed generated in 10% FBS and at a certain concentration that could inhibit cell growth.

### Effects of GROs and dG on cell cycle and apoptotic profile

Some GROs have been reported to induce apoptosis in tumor cells^21^,^47^,^48^ and induce the accumulation of cells in S phase and in sub-G1 phase.^20^,^41^,^47^ In order to further compare the effects of guanine-based compounds and GROs on cell cycle and apoptotic profile, we performed Annexin V-fluorescein assay and cell cycle assay after treating cells with 10 μM TT-loop, de-TT-loop (TT-loop pretreated in 50% serum for 48 h), AS1411 or different concentrations of dG for different times.

**Fig. 6:**
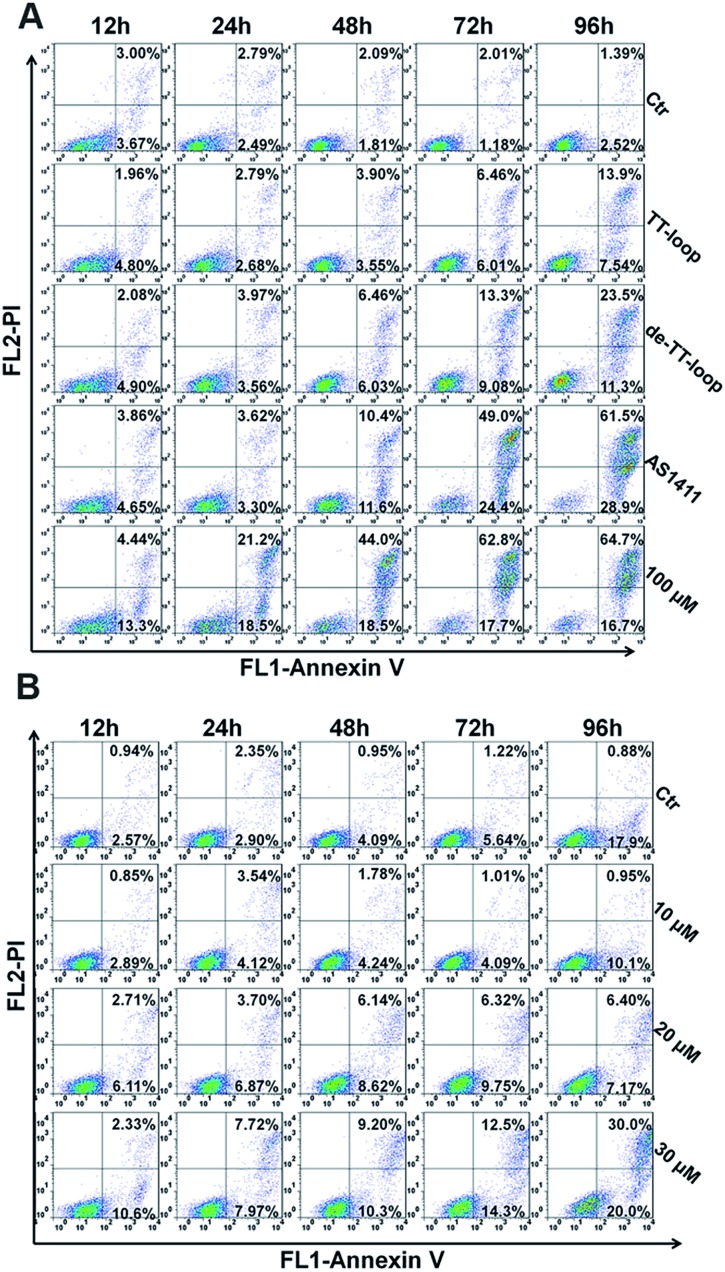
Cell apoptosis and death induced by GROs and dG. (A) Jurkat E6-1 cells were treated with 10 μM TT-loop, de-TT-loop (pre-degraded in 50% FBS for 48 h), AS1411 or 100 μM dG for 12, 24, 48, 72 and 96 h. (B) Jurkat E6-1 cells were treated with 10, 20 and 30 μM dG for 6, 12, 24, 48, 72 and 96 h. Cells were double-stained by Annexin V-FITC and PI. Crossing gates divided the dot plots into four quadrants. Dots in the lower right quadrants (Annexin V+ and PI–) indicate early apoptotic cells; dots in upper right quadrants (Annexin V+ and PI+) indicate late apoptotic cells or dead cells. The numbers indicate the percentage of cells in the corresponding quadrants. Ctr: cells without treatment. The result is a single representative of three independent experiments.

The apoptotic profiles of cells were measured by flow cytometry. Compared with the untreated cells (control), all the treatments were observed to induce apoptosis and the death of Jurkat E6-1 cells, but different treatments showed different time dependent profiles (Fig. 6A). TT-loop treatment only induced apoptosis and death of a small fraction of cells from 72 h (∼12%) to 96 h (∼21%); the pre-degraded TT-loop (de-TT-loop) treatment induced notable apoptosis and death of cells from 48 h (∼12%) to 96 h (∼35%); AS1411 treatment caused a larger population of apoptotic and dead cells (∼22%) in 48 h than de-TT-loop treatment and caused the death of most cells in 96 h (∼90%); 100 μM dG (equal to 8.3 μM TT-loop in guanine) treatment caused ∼18% cell apoptosis and death as early as 12 h, caused 40–63% cell apoptosis and death from 24 to 48 h and ∼80% cell apoptosis and death in 72 h. These results suggest that dG (100 μM) had the strongest toxicity to Jurkat E6-1 cells. AS1411 (10 μM) showed similar cytotoxicity with dG after 72 h treatment, but the toxicity occurred slower than that of dG, which may due to the delayed release of guanine-based degradation products. TT-loop showed the weakest cytotoxicity because of its low degradation rate, which can be further confirmed by the faster and stronger cytotoxicity of the de-TT-loop than the TT-loop.

Since the high concentration of dG caused strong cytotoxicity, we also measured the apoptotic profile of cells treated with lower concentrations of dG. 10 μM dG did not cause notable apoptosis and death of Jurkat E6-1 cells even after 96 h. 20 and 30 μM dG caused a small population of apoptosis and death of cells (Fig. 6B), which was similar with that of the TT-loop (10 μM) and the de-TT-loop (10 μM). However, the microscopic observation of cell growth in the presence of dG showed that 10 μM dG did not affect the cell growth even after 96 h, but 20 and 30 μM dG greatly inhibited the cell growth (Fig. S5†), which was consistent with the antiproliferative effect of dG (Fig. 2C). This set of results suggests that 20–30 μM dG mainly inhibited the cell growth and only induced apoptosis and the death of a small amount of cells.

**Fig. 7:**
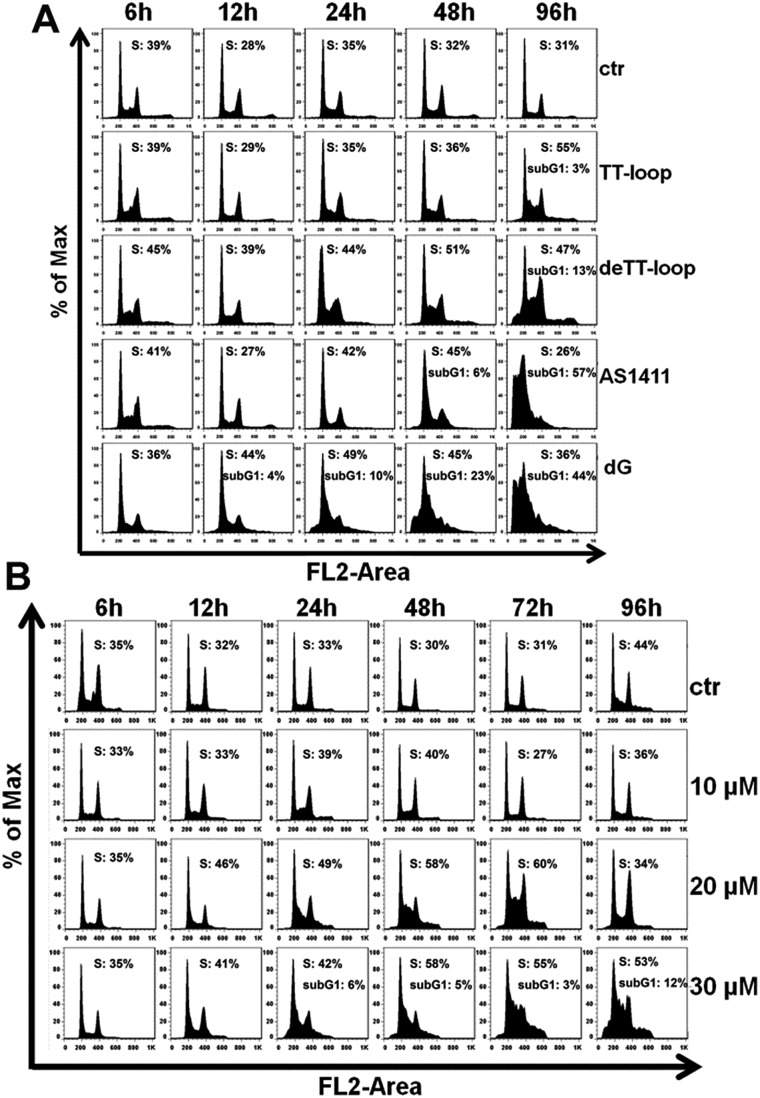
Cell cycle profile analysis of Jurkat E6-1 cells treated by GROs and dG. (A) Cell-cycle phase distribution of cells treated with 10 μM TT-loop, de-TT-loop, AS1411 or 100 μM dG at 6, 12, 24, 48 and 96 h. (B) Cell-cycle phase distribution of cells treated with 10, 20 or 30 μM dG at 6, 12, 24, 48, 72 and 96 h. Quantification of S phase and sub-G1 phase was performed by FlowJo (Treestar, San Caros, USA). Ctr: cells without treatment. The result is a single representative of three independent experiments.

The results of the cell cycle assay are shown in Fig. 7. Similar to previous reports,^20^,^41^,^47^ GROs or dG treatment was also found to increase the cell population in the S- and sub-G1 phases. Cell population in the sub-G1 phase is indicative of apoptotic and dead cells. TT-loop treatment was found to cause an increase of cells in the S-phase and sub-G1 phase at 96 h; while de-TT-loop treatment caused significant increase of cells in the S-phase from 24 to 96 h and a larger population of cells in the sub-G1 phase at 96 h than TT-loop treatment. AS1411 treatment caused the appearance of cells in the sub-G1 phase at 48 h, and caused 57% of cells in the sub-G1 phase at 96 h, which was consistent with the observation by Xu and coauthors.^41^ 100 μM dG treatment caused the appearance of the sub-G1 phase population as early as 12 h, and caused 44% cells in the sub-G1 phase at 96 h. But 10 μM dG did not cause a notable cell cycle change even after 96 h. 20 and 30 μM dG caused a significant increase of cells in the S-phase from 12 to 96 h. 30 μM dG treatment also caused the appearance of cells in the sub-G1 phase from 24–96 h. This set of results agreed well with the apoptotic profiles (Fig. 6) and also suggests that GROs may have a similar mechanism of action to dG.

## Discussion

Based on the above results, we are confident that the antiproliferative activity of GROs is mainly contributed by the cytotoxicity of their degradation products, *i.e.* dGMP, dG and guanine. This conclusion is drawn from the following three aspects:

i) G-quadruplex oligonucleotides with very high thermostability and nuclease resistance (T-loop, C-loop, C3-loop and S9-loop) showed very weak antiproliferative effects. G-quadruplex oligonucleotides with relatively lower thermostability and nuclease resistance (A-loop, TT-loop, CC-loop, AA-loop, HH-loop, TTT-loop and HHH-loop) and GROs that cannot form intramolecular G-quadruplex (H-G4 and G-control) showed significant antiproliferative activity. AS1411 with the lowest nuclease resistance showed the highest antiproliferative activity. These results imply that the antiproliferative activity of GROs does not relate to the G-quadruplex structures, but relates to their nuclease resistance.

ii) Oligonucleotides without guanine base did not show antiproliferative effects. Among nucleotides, nucleosides and nucleobases, only guanine-based compounds showed antiproliferative activity. After incubation of AS1411 or TT-loop with 10% FBS, guanine-based compounds were detected, and their concentrations were enough to inhibit cell growth. TT-loop, AS1411 and dG showed parallel antiproliferative effects on seven cell lines. These results indicate that guanine-based degradation products must have contributed to the antiproliferative effect of GROs.

iii) The cell cycle and apoptotic profiles assay showed that GROs exhibited delayed effects (apoptosis and death) compared with dG. The pre-degraded TT-loop (de-TT-loop) showed faster effects than the TT-loop. AS1411 that had a high degradation rate showed a strong cytotoxicity similar with that of the high concentration of dG (100 μM). The TT-loop that had a lower degradation rate only induced a small population of apoptotic and dead cells, which was similar with that of the low concentration of dG (20 μM). These time-related and degradation rate-related effects confirm that the antiproliferative effect of GROs is mainly contributed by their degradation products, not by the GROs themselves.

As endogenous molecules, nucleotides, nucleosides and nucleobases not only serve as substrates for nucleic acid biosynthesis but also participate in the energy metabolism and signal transduction. In addition to the wide range of biological activities under both physiological and pathological conditions, the cytotoxicity of guanine-based nucleotides and nucleosides to several cancer cell lines have been reported over the past three decades.^49^–^55^ However, not much attention has been paid to the cytotoxicity of guanine-based nucleotides, nucleosides and guanine, which may be because their cytotoxicity is diverse and depends on specific cells. Besides, they are endogenous compounds, and their cytotoxicity is usually observed at higher concentrations (>50 μM). Although some mechanisms of action of guanine-based compounds have been proposed,^49^–^55^ the exact mechanism remains unclear. Our results show that the cytotoxicity of guanine-based compounds is highly dependent on their concentration. Their IC_50_ values to Jurkat E6-1 cells were 14–18 μM. At concentrations less than 10 μM, dG did not show any effects to Jurkat E6-1 cells. 20–30 μM dG mainly inhibited cell growth and did not significantly induce cell apoptosis and death. High concentrations of dG (100 μM) exhibited strong cytotoxicity.

Conversely the antiproliferative activity of GROs has attracted extensive attention in recent years. For the most part, GROs have been shown to form G-quadruplex structures.^20^–^25^ G-quadruplex-forming sequences have been reported to be highly prevalent in the genome, as well as in particular RNA domains.^56^–^59^ Accumulating evidence suggests that G-quadruplexes play important roles *in vivo* in regulating gene expression and telomere stability.^11^,^60^ There is no doubt that the biological functions of G-quadruplexes need the participation of many G-quadruplex-binding proteins in cells, although only a few of them have been identified.^11^,^12^,^23^,^24^,^29^,^59^,^61^ Therefore, the antiproliferative effect of extraneous GROs is considered to result from their binding to G-quadruplex-binding proteins, thus causing disturbance to the expression and regulation of G-quadruplex related genes. If this is the case, the GROs with good cellular uptake and nuclease resistance would have high antiproliferative activity.

Reports concerning the cellular uptake of GROs are quite common, but systematic studies are few.^12^ In general, the GROs with higher nuclease resistance are found to have higher cellular uptake.^13^,^42^ However, the above results show that GROs with high nuclease resistance have low antiproliferative activity. Although GROs with low nuclease resistance were also found in cells, many of the uptake studies are performed by measuring the fluorescence in cells after being treated with dye-labelled GROs,^39^,^42^ this method cannot really indicate that the fluorescence was from the intact GRO, degraded GRO, or the dye cleaved from GRO, especially for GROs with low nuclease resistance. To date, the mechanism of cellular uptake of synthetic GROs is rather poorly understood. Many researchers believe that receptor (*e.g.* cell surface nucleolin) mediated endocytosis is the predominant mechanism,^12^,^39^,^62^ but different mechanisms such as micropinocytosis have also been proposed.^39^ In a previous study, we have observed that the cellular uptake and antiproliferative activity of GROs is independent of their cellular binding.^42^ All the contradictory results suggest that the antiproliferative activity of GROs may not be mainly contributed by the internalized GROs.

Indeed, G-quadruplex oligonucleotides show enhanced resistance to serum nuclease compared with other non-quadruplex oligonucleotides, which usually delay their degradation from several minutes to several hours, completely degrading in a longer time as shown in our results. Usually, the antiproliferative investigations were performed in 72–120 h after GRO treatment,^20^,^26^,^30^,^41^,^48^ in this time period, the cytotoxicity of the degradation products cannot be neglected. However, it is still possible that the enhanced biostability and cellular uptake may provide G-quadruplex oligonucleotides the chance to bind to their target proteins in cells and disturb the cell functions. Therefore we cannot completely exclude the possibility that some G-quadruplex oligonucleotides themselves contribute to their antiproliferative activity. But we believe that the toxicity of guanine-based degradation products largely contributes to the antiproliferative activity of GROs, especially the nuclease sensitive GROs.

AS1411 has reached the phase II trial stage as an anticancer reagent. It has been reported to display antiproliferative activity in almost 80 tumor cell lines, and the typical IC_50_ values are in the range of 1–10 μM 12,34,41 which correspond to 17–170 μM dG. In the phase II clinical studies, it was administered at a high dosage (40 mg per kg per day) by continuous intravenous infusion.^12^,^32^ In addition, AS1411 does not cause rapid cytotoxicity, the inhibition of cell growth and induction of cell death usually occurs after prolonged exposure to AS1411 (2–4 days),^12^,^33^ this is why a continuous infusion of AS1411 for 4 or 7 days is chosen as the route of administration for clinical studies.^12^,^32^ Our results have shown that most of AS1411 were digested in cell culture medium over several hours and 27–74% of them were converted to guanine-based compounds in 6–48 h in 10% FBS solution. Compared to dG, AS1411 showed a delayed cytotoxicity. Therefore, It can be concluded that the biological activity of AS1411 mainly be due to the action of its guanine-based degradation products.

## Conclusions

In summary, we have provided solid evidence that the antiproliferative activity of GROs was mainly contributed by the cytotoxicity of their guanine-based degradation products. We also showed the highly dose-dependent cytotoxicity of guanine-based compounds. These results suggest that systematic studies of the cytotoxicity of guanine-based compounds and their mechanism of action will provide a deep insight into the function of guanine-based compounds and offer useful information for drug design.

## Supplementary Material

Supplementary information

## References

[cit1] Kole R., Krainer A. R., Altman S. (2012). Nat. Rev. Drug Discovery.

[cit2] Keefe A. D., Pai S., Ellington A. (2010). Nat. Rev. Drug Discovery.

[cit3] Sundaram P., Kurniawan H., Byrne M. E., Wower J. (2013). Eur. J. Pharm. Sci..

[cit4] Ye M., Hu J., Peng M., Liu J., Liu J., Liu H., Zhao X., Tan W. (2012). Int. J. Mol. Sci..

[cit5] Zhu G., Ye M., Donovan M. J., Song E., Zhao Z., Tan W. (2012). Chem. Commun..

[cit6] Fang X., Tan W. (2010). Acc. Chem. Res..

[cit7] Vollmer J., Krieg A. M. (2009). Adv. Drug Delivery Rev..

[cit8] Soldati C., Bithell A., Conforti P., Cattaneo E., Buckley N. J. (2011). J. Neurochem..

[cit9] Juliano R. L., Ming X., Carver K., Laing B. (2014). Nucleic Acid Ther..

[cit10] Juliano R. L., Ming X., Nakagawa O. (2012). Bioconjugate Chem..

[cit11] Collie G. W., Parkinson G. N. (2011). Chem. Soc. Rev..

[cit12] Bates P. J., Laber D. A., Miller D. M., Thomas S. D., Trent J. O. (2009). Exp. Mol. Pathol..

[cit13] Bishop J. S., GuyCaffey J. K., Ojwang J. O., Smith S. R., Hogan M. E., Cossum P. A., Rando R. F., Chaudhary N. (1996). J. Biol. Chem..

[cit14] Cao Z. H., Huang C. C., Tan W. H. (2006). Anal. Chem..

[cit15] Bartz H., Mendoza Y., Gebker M., Fischborn T., Heeg K., Dalpke A. (2004). Vaccine.

[cit16] Bock L. C., Griffin L. C., Latham J. A., Vermaas E. H., Toole J. J. (1992). Nature.

[cit17] Jing N., Marchand C., Liu J., Mitra R., Hogan M. E., Pommier Y. (2000). J. Biol. Chem..

[cit18] Suzuki J.-i., Miyano-Kurosaki N., Kuwasaki T., Takeuchi H., Kawai G., Takaku H. (2002). J. Virol..

[cit19] Fennewald S. M., Mustain S., Ojwang J., Rando R. F. (1995). Antiviral Res..

[cit20] Rankin A. M., Forman L., Sarkar S., Faller D. V. (2013). Nucleic Acid Ther..

[cit21] Qi H., Lin C.-P., Fu X., Wood L. M., Liu A. A., Tsai Y.-C., Chen Y., Barbieri C. M., Pilch D. S., Liu L. F. (2006). Cancer Res..

[cit22] Yaar M., Eller M. S., Panova I., Kubera J., Wee L. H., Cowan K. H., Gilchrest B. A. (2007). Breast Cancer Res..

[cit23] Jing N., Li Y., Xiong W., Sha W., Jing L., Tweardy D. J. (2004). Cancer Res..

[cit24] Jing N., Zhu Q., Yuan P., Li Y., Mao L., Tweardy D. J. (2006). Mol. Cancer Ther..

[cit25] Weerasinghe P., Garcia G. E., Zhu Q., Yuan P., Feng L., Mao L., Jing N. (2007). Int. J. Oncol..

[cit26] Choi E. W., Nayak L. V., Bates P. J. (2010). Nucleic Acids Res..

[cit27] Goodchild A., King A., Gozar M. M., Passioura T., Tucker C., Rivory L. (2007). Nucleic Acids Res..

[cit28] Scaggiante B., Dapas B., Grassi G., Manzini G. (2006). FEBS J..

[cit29] Aoki H., Iwado E., Eller M. S., Kondo Y., Fujiwara K., Li G.-Z., Hess K. R., Siwak D. R., Sawaya R., Mills G. B., Gilchrest B. A., Kondo S. (2007). FASEB J..

[cit30] Soundararajan S., Chen W. W., Spicer E. K., Courtenay-Luck N., Fernandes D. J. (2008). Cancer Res..

[cit31] Guan Y., Reddy K. R., Zhu Q., Li Y., Lee K., Weerasinghe P., Prchal J., Semenza G. L., Jing N. (2010). Mol. Ther..

[cit32] Rosenberg J. E., Bambury R. M., Van Allen E. M., Drabkin H. A., Lara P. N., Harzstark A. L., Wagle N., Figlin R. A., Smith G. W., Garraway L. A., Choueiri T., Erlandsson F., Laber D. A. (2014). Invest. New Drugs.

[cit33] Ireson C. R., Kelland L. R. (2006). Mol. Cancer Ther..

[cit34] Girvan A. C., Teng Y., Casson L. K., Thomas S. D., Juliger S., Ball M. W., Klein J. B., Pierce W. M., Barve S. S., Bates P. J. (2006). Mol. Cancer Ther..

[cit35] Li L., Hou J., Liu X., Guo Y., Wu Y., Zhang L., Yang Z. (2014). Biomaterials.

[cit36] Li J., Zheng H., Bates P. J., Malik T., Li X.-F., Trent J. O., Ng C. K. (2014). Nucl. Med. Biol..

[cit37] Xing H., Tang L., Yang X., Hwang K., Wang W., Yin Q., Wong N. Y., Dobrucki L. W., Yasui N., Katzenellenbogen J. A., Helferich W. G., Cheng J., Lu Y. (2013). J. Mater. Chem. B.

[cit38] Qiu L. P., Wu C. C., You M. X., Han D., Chen T., Zhu G. Z., Jiang J. H., Yu R. Q., Tan W. H. (2013). J. Am. Chem. Soc..

[cit39] Reyes-Reyes E. M., Teng Y., Bates P. J. (2010). Cancer Res..

[cit40] Teng Y., Girvan A. C., Casson L. K., Pierce W. M., Qian N., Thomas S. D., Bates P. J. (2007). Cancer Res..

[cit41] Xu X., Hamhouyia F., Thomas S. D., Burke T. J., Girvan A. C., McGregor W. G., Trent J. O., Miller D. M., Bates P. J. (2001). J. Biol. Chem..

[cit42] Chang T., Qi C., Meng J., Zhang N., Bing T., Yang X., Cao Z., Shangguan D. (2013). PLoS One.

[cit43] Smargiasso N., Rosu F., Hsia W., Colson P., Baker E. S., Bowers M. T., De Pauw E., Gabelica V. (2008). J. Am. Chem. Soc..

[cit44] Guedin A., Gros J., Alberti P., Mergny J.-L. (2010). Nucleic Acids Res..

[cit45] Cheng X. H., Liu X. J., Bing T., Zhao R., Xiong S. X., Shangguan D. H. (2009). Biopolymers.

[cit46] Zhou W., Chen Q., Huang P. J., Ding J., Liu J. (2015). Anal. Chem..

[cit47] Schwartz T. R., Vasta C. A., Bauer T. L., Parekh-Olmedo H., Kmiec E. B. (2008). Oligonucleotides.

[cit48] Sarkar S., Faller D. V. (2013). Nucleic Acid Ther..

[cit49] Batiuk T. D., Schnizlein-Bick C., Plotkin Z., Dagher P. C. (2001). Am. J. Physiol.: Cell Physiol..

[cit50] Duan D. S., Nagashima T., Hoshino T., Waldman F., Pawlak K., Sadee W. (1990). Biochem. J..

[cit51] Cohen A., Lee J. W., Gelfand E. W. (1983). Blood.

[cit52] Moosavi M. A., Yazdanparast R., Lotfi A. (2007). Int. J. Biochem. Cell Biol..

[cit53] Guarnieri S., Pilla R., Morabito C., Sacchetti S., Mancinelli R., Fano G., Mariggio M. A. (2009). Int. J. Dev. Neurosci..

[cit54] Sidi Y., Mitchell B. S. (1984). J. Clin. Invest..

[cit55] Flanagan S. A., Gandhi V., Meckling K. A. (2007). Leuk. Lymphoma.

[cit56] Huppert J. L., Balasubramanian S. (2005). Nucleic Acids Res..

[cit57] Todd A. K., Johnston M., Neidle S. (2005). Nucleic Acids Res..

[cit58] Agarwala P., Pandey S., Maiti S. (2014). Biochim. Biophys. Acta, Gen.
Subj..

[cit59] Xu Y. (2011). Chem. Soc. Rev..

[cit60] Balasubramanian S., Hurley L. H., Neidle S. (2011). Nat. Rev. Drug Discovery.

[cit61] Sanders C. M. (2010). Biochem. J..

[cit62] Bates P. J., Kahlon J. B., Thomas S. D., Trent J. O., Miller D. M. (1999). J. Biol. Chem..

