# Supplementary material for: Cytotoxicity of guanine-based degradation products contributes to the antiproliferative activity of guanine-rich oligonucleotides
Source: Chem Sci. 2015 Apr 7;6(7):3831–8. doi: 10.1039/c4sc03949a (PMC5707456; doi:10.1039/c4sc03949a)
Supplement: Supplementary file 1 [file SC-006-C4SC03949A-s001.pdf]

Electronic Supplementary Material (ESI) for Chemical Science.
This journal is © The Royal Society of Chemistry 2015

Supplementary information

Cytotoxicity of Guanine-Based Degradation Products Contributes to the Antiproliferative Activity of Guanine-rich Oligonucleotides

Nan Zhang[1,2], Tao Bing[1], Xiangjun Liu[1], Cui Qi[1], Luyao Shen[1,2], Linlin Wang[1], & Dihua Shangguan[1*]

[1] Beijing National Laboratory for Molecular Sciences, Key Laboratory of Analytical Chemistry for Living Biosystems, Institute of Chemistry, Chinese Academy of Sciences, Beijing, 100190, china.

[2] University of the Chinese Academy of Sciences, Beijing 100049, China

*** Corresponding Author:**

Tel&FAX：86-10-62528509, E-mail: sgdh@iccas.ac.cn (Dr. D. Shangguan)

Results

CD Spectra of Guanine-rich Oligonucleotides

CD spectra can provide information concerning the structure of G-quadruplexes: the anti-parallel G-quadruplexes exhibit a positive ellipticity maximum around 295 nm and a negative minimum around 260 nm; the parallel G-quadruplexes exhibit a positive maximum around 264 nm and a negative minimum around 240 nm; and the mixed parallel/anti-parallel structures show a positive maximum around 295nm plus a positive signal near 265 nm and a negative minimum at 235-240 nm1. As shown in **Fig.S1**, the oligonucleotides with single-base loops (T-loop, C-loop, A-loop and H-loop) and the oligonucleotides with non-nucleotide loops (C3-loop and S9-loop) exhibited strong CD signal of parallel G-quadruplexes, suggesting that these oligonucleotides fold into parallel G-quadruplexes with high stability. The oligonucleotides with two-base loops (TT-loop, AA-loop, CC-loop and HH-loop)

exhibited weak characteristic signals of hybrid or mixed parallel G-quadruplexes except for TT-loop who exhibited strong CD signals. The oligonucleotides with three-base loops (TTT-loop and HHH-loop) exhibited very weak CD signals of hybrid or mixed parallel G-quadruplexes. AS1411 showed characteristic signals of hybrid or mixed G-quadruplexes. These results confirmed that the tested oligonucleotides could fold into G-quadruplexes. Oligonucleotides C-control and G-control did not show characteristic CD signals of G-quadruplexes. Oligonucleotide H-G4 showed weak CD signal of parallel G-quadruplexes, suggesting that it might formed intermolecular G-quadruplex.

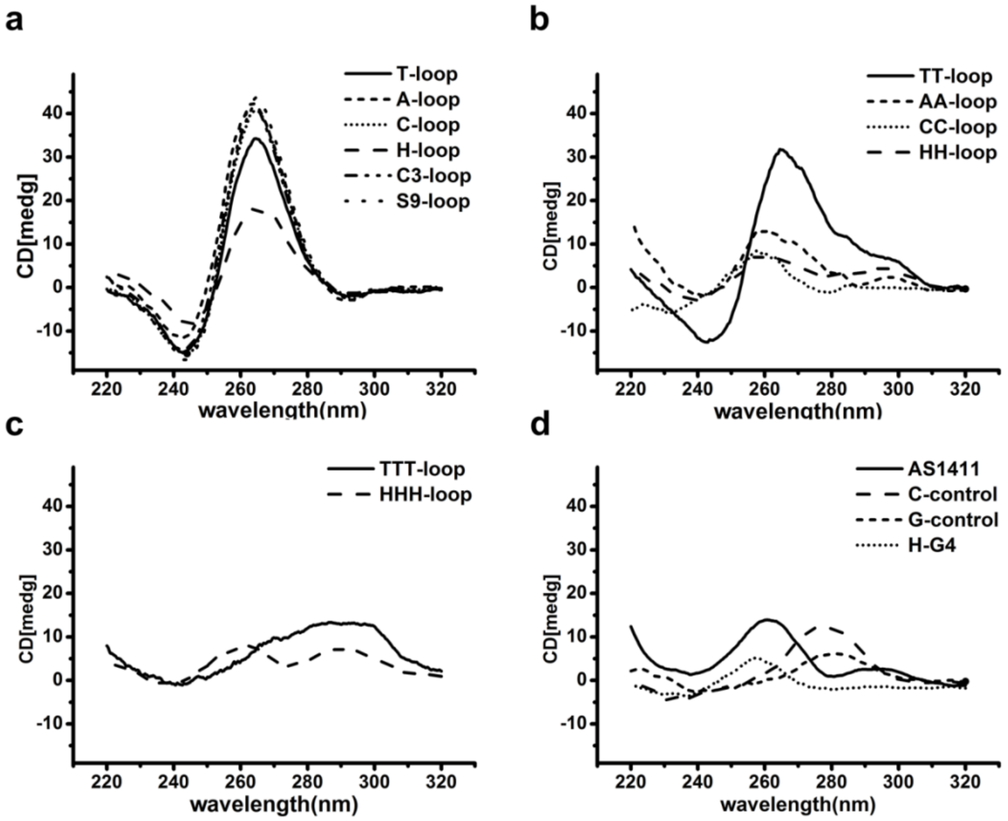

Fig. S1. CD spectra of DNA oligonucleotodes. All oligonucleotides were dissolved in PBS buffer, pH=7.4 at a final concentration of 4 μM.

The correlation of degradation rate and cytotoxicity of GROs

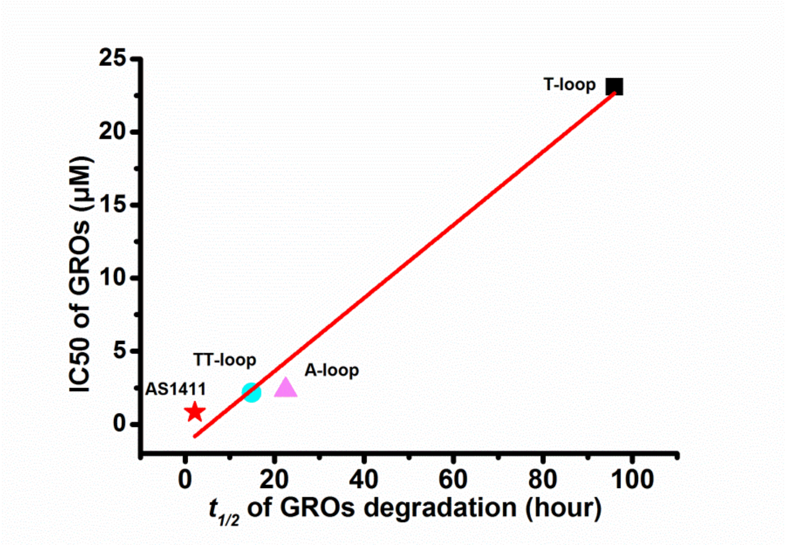

Fig. S2 The correlation plot of IC_{50} of GRQs for Jurkat cells VS half-life of GROs in 10% FBS.

HPLC analysis of guanine-based degradation products

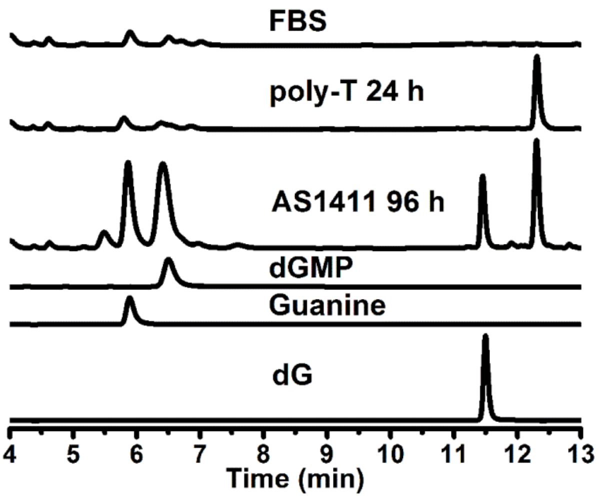

Fig. S3. HPLC chromatogram of dGMP, dG, guanine and degradation products of AS1411 and poly-T.

AS1411 (10 μM) or poly-T (poly-T_{10}, 20 μM) were incubated in 800 μL of PBS containing 10% FBS at 37°C. 100 μL of incubated sample was taken out atdifferent times, and added with 300 μL of acetonitrile for protein-precipitation, after

centrifuging (12000 rpm, 5 min) the supernatants were dried and stored at -20°C before analysis. Samples were dissolved in 20 uL of mobile phase B, and 10 μL of sample was applied for reversed phase-HPLC assay. The standard samples (100 μM dGMP, dG and guanine) were dissolved in mobile phase B; 10 μL of sample was applied for reversed phase-HPLC assay. HPLC conditions: C18 column, Agela, 5 μm, 100Å, 4.6×250 mm; mobile phase A, Acetonitrile; mobile phase B, 20mM KH_2PO_4/Et_3N buffer (pH=6.5); the gradient program: 0-2min, 3% A； 2-15min, 3%-15% A; flow rate: 1 ml/min, UV detection at 260 nm.

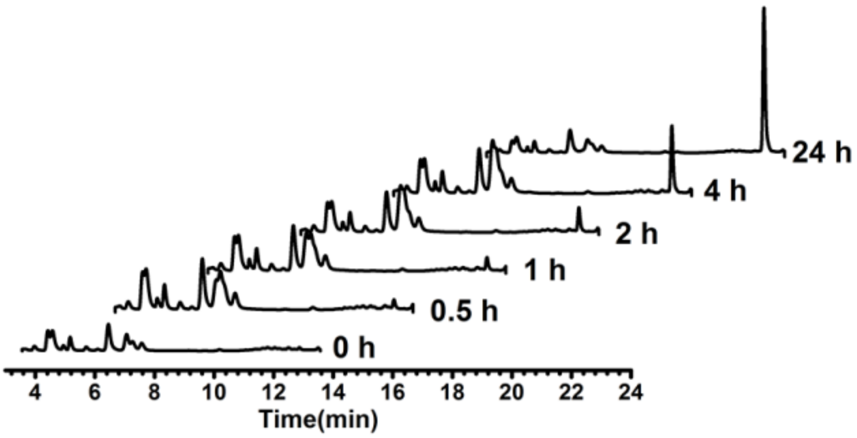

Fig. S4. HPLC analysis of the degradation products of poly-T oligonucleotides.

Poly-T (T_{10}, 20 μM) was incubated with 10% FBS in PBS at 37 ^{o}C for different times. poly-T (poly-T_{10}, 20 μM) were incubated in 800 μL of PBS containing 10% FBS at 37°C. 100 μL of incubated sample was taken out at different times, and added with 300 μL of acetonitrile for protein-precipitation, after centrifuging (12000 rpm, 5 min) the supernatants were dried and stored at -20°C before analysis. Samples were dissolved in 20 uL of mobile phase B, and 10 μL of sample was applied for reversed phase-HPLC assay. The HPLC conditions are identical to **Supplementary figure 2.**

Growth observation of Jurkat E6-1 cells after treated by dG

Although Jurkat E6-1 is a suspension cultured cell line, the cells prefer to form aggregates when they grow to high density. As shown in **Figure S4,** the cell density and the aggregate size of untreated cells (control) increased with time of culture;

similar results was also observed in cells treated with 10 μM dG, indicating that 10 μM dG did not affect cell growth much. However, after treated with 20 μM dG, the cell growth was significantly inhibited, only some small size aggregates were observed after 96 h. After treated with 30 μM dG, the cell growth was totally inhibited, no notable aggregates were observed after 96 h.

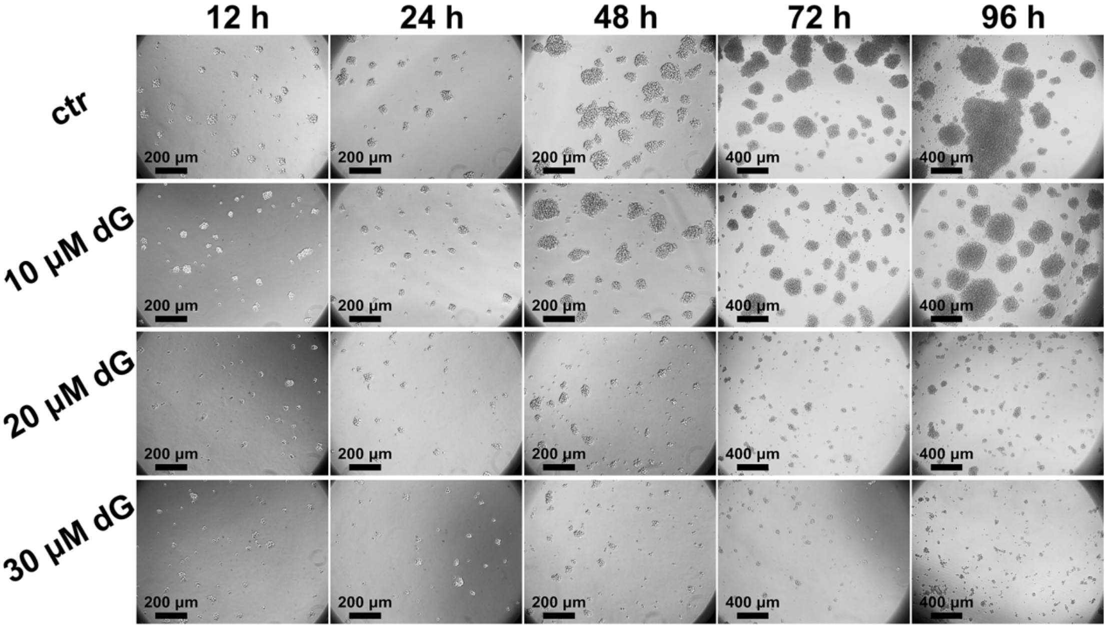

Fig. S5. The densities and aggregation states of Jurkat E6-1 after treated by dG for different times. The pictures were taken on a fluorescent microscope (Olympus IX71S1F3, Japan)

Materials and methods

Oligonucleotides. All oligonucleotides (**Table 1**) were synthesized by Sangon Biotech (Shanghai, China); and to ensure the results' reliability, some oligonucleotides were resynthesized by Sunbiotech Co., Ltd (Beijing, China) to duplicate the experiments. The stock solutions were prepared by dissolving the freeze-dried oligonucleotides in PBS and stored at -20°C. The concentrations were measured based on their absorbance at 260 nm and their molar extinction coefficients. Unless otherwise specified, before used all oligonucleotides were heat-denatured at 95°C for 5 min, and then placed on the ice for 10 min, annealed at room temperature for 2-4 h

and kept at 4°C overnight to form stable secondary structures.

Cell Culture. Jurkat E6-1 (Acute T lymphoblastic leukemia) and K562 (Chronic myelogenous leukemia) cell lines were purchased from Cell Culture Center of Insititute of Basic Medical Sciences, Chinese Academy of Medical Sciences (Beijing, China). A549 (non-small cell lung cancer) and MCF-7 (breast cancer) cell lines were purchased from Cell Resource Center of Shanghai Institute for Biological Sciences (Chinese Academy of Sciences, Shanghai, China). DU145 (prostate cancer, derived from brain metastasis) and PC-3 (prostate cancer) cell lines were purchased from typical culture preservation commission cell bank, Chinese Academy of Sciences (Shanghai, China). A549T (Taxol-resistant A549 subline) cell line was purchased from Shanghai Aiyan Biological Technology Co. Ltd (Shanghai, China).

Unless otherwise stated, the basic media were supplemented with 10% fetal bovine serum (FBS, Gibco) and 1% penicillin/streptomycin (Corning). Jurkat E6-1, K562, DU145, PC-3 and A549T are cultured in RPMI-1640 medium (Corning). A549 and MCF-7 were propagated in Dulbecco's Modified Eagle Medium (DMEM, Corning). All cell lines were routinely cultured at 37°C in humidified atmosphere with 5% CO_2.

Circular Dichroism (CD) Spectra measurement

Oligonucleotides were diluted to a concentration of 4 μM by PBS，and stabilized at room temperature for 2-4 h. The CD spectra (220-320 nm) were collected at room temperature on Jasco J-815 spectropolarimeter (Japan). The scanning rate was 500 nm/min. Each sample was scaned three times and the mean value was taken. To facilitate analysis, all CD spectra were applied background subtraction and smoothed.

Oligonucleotides degradation in serum-containing medium. For high sensitive detection, oligonucleotides used in this experiment were labeled with fluorescein (FAM) at 5' end. After annealed, 10 μM oligonucleotides were added into RPMI 1640 medium with 10% FBS and incubated in a humidified 37°C/5% CO_2 incubator. Aliquots (20 μL) of the incubated samples was taken out at 0, 6, 12, 24, 48 and 96 h, added with 25 mM EDTA, and heated at 95°C for 5 min to quench the nuclease, and then stored at -20°C before analysis. 20% denaturing-polyacrylamide gel electrophoresis was used to analyze these urea-denaturing samples. The gels were exposed under UV light and photographed. The fluorescence intensities (quantified by

Image J (NIH, USA)) of the major bands corresponding to intact DNAs were used to evaluate the stability of the FAM- labeled oligonucleotides.

HPLC analysis of guanine-based degradation products.

AS1411 (10 μM), TT-loop (10 μM) or poly T_{10} (poly-T, 20 μM) were incubated in 800 μL of PBS containing 10% FBS at 37°C. 100 μL of incubated sample was taken out at 0, 6, 12, 24, 48, 72 and 96 h, and added with 300 μL of acetonitrile for protein-precipitation, after centrifuging (12000 rpm, 5 min) the supernatants were dried and stored at -20°C before analysis. Samples were dissolved in 20 uL of 20 mM KH_2PO_4/Et_3N buffer pH 6.5, and 10 μL of sample was applied for reversed phase-HPLC assay. Condition：C_{18} column: Agela, 5 μm, 100Å, 4.6×250 mm; mobile phase A: Acetonitrile, mobile phase B: 20mM KH_2PO_4/Et_3N buffer (pH 6.5); the gradient program: 0-2 min, 3% A；2-15 min, 3%-15% A; flow rate: 1 mL/min, UV detection at 260 nm.

Cell proliferation Assay.

Cells were seeded in 96-well plates (100 μL per well). Different cell lines were seeded at different densities and pre-incubated for different time due to their intrinsic different growth rate. Jurkat and K562 were seeded at 3000/well and pre-incubated 4 h. A549, PC-3, and DU145 were seeded at 1000/well and pre-incubated overnight. A549T and MCF-7 were seeded at 1000/well and pre-incubated 4 h. The oligonucleotides, nucleosides, nucleotides or nucleobases were then added into each well to get a desired final concentration. To ensure the results' reliability, each concentration was repeated three wells and each experiment independently performed at least twice. Cells in 96-well plates were incubated for desired time (usually 96 h) without changing medium. Cell viability was measured by Cell Counting Kit-8 (CCK-8, Dojindo, Japan) assay according to the guidelines recommended. 10 μL of CCK-8 was added into each well and incubated with cells for 1-4 h. The absorbance was measured on a microplate reader (Spectra Max M5, Molecular Device Co.) at 450 nm. Control samples incubated with PBS instead of oligonucleotides. Cell viability was obtained by comparing the absorbance of treated cells to that of control cells.

Cell apoptosis assay

Jurkat cells were seeded at a density of 1×10^5 /mL. After pre-incubation for 4 h, cells were added with oligonucleotides (10 μM) or dG (10, 20, 30 and 100 μM) and

cultured. Aliquots of treated cells were taken for analysis at time points of 6, 12, 24, 48, 72 and 96 h. After washed, cells were stained with Annexin V-FITC for 30 min at room temperature in the dark and then stained by PI on the ice, and then were measured by flow cytometry with FL1 and FL2 channels. Annexin V-FITC Apoptosis Detection Kit was purchased from Dojindo Co. Ltd (Japan). Quantification of S phase and sub-G1 phase was done by FlowJo (Treestar, San Caros, USA).

Cell cycle profiles analysis

Jurkat cells were seeded at a density of 1×10^5/mL in 48-well plates. After pre-incubation for 4 h, cells were added with oligonucleotides (10 μM) or dG (10, 20, 30 and 100 μM) and cultured. Aliquots of treated cells were taken for analysis at time points of 6, 12, 24, 48, 72 and 96 h. Cells were centrifuged and re-suspended in 50 μL ice-cold PBS, fixed by addition of 500 μL of 78% ethanol, and stored at -20°C at least overnight. After fixed, cells were washed twice with ice cold PBS and re-suspended in 400 μL of PI staining buffer (PBS containing 50 μg/mL propidium iodide (PI)，10 μg/mL RNase A and 0.5% Triton-X-100) for 30 min at room temperature in the dark. The DNA content was acquired by a Becton Dickinson FACScalibur flow cytometer (Becton Dickinson, USA) with FL2 channel. The data were analyzed by FlowJo software (Treestar, San Caros, USA).

References

1. Burge, S.; Parkinson, G. N.; Hazel, P.; Todd, A. K.; Neidle, S., Quadruplex DNA: sequence, topology and structure. *Nucleic Acids Res* **2006,** *34* (19), 5402-15.
